# Study on the Dynamic Compound Structure Composed of Mast Cells, Blood Vessels, and Nerves in Rat Acupoint

**DOI:** 10.1155/2013/160651

**Published:** 2013-06-26

**Authors:** Luo Mingfu, Dong Xiaotong, Song Xiaojing, Jiang Jin, Zhann Jinling, Han Ying

**Affiliations:** Institute of Acupuncture and Moxibustion, China Academy of Chinese Medical Sciences, Beijing 100700, China

## Abstract

*Background*. Circulation system, immunity system, and nervous system have a close relationship with meridian phenomen. However, there is still lack of the results of dynamic changes of these structures in acupoint. The aim of this study is to explore the interrelationship by composite staining techniques. *Methodology/Principal Findings*. Twenty rats were separated into electroacupuncture group (EA) and control group (Con) randomly. In EA group, the Zusanli and Weishu were stimulated with the 0.1 mA for 25 min. The tissue of these acupoints was double-stained with acetylcholinesterase and Toluidine blue. The compound structure of mast cells, nervous fibers, and mast cells in the acupoint was observed. *Conclusions/Significance*. The blood vessels, mast cells and acetylcholinesterase responded nerves were clearly observed in acupoint tissues. EA can result in the mast cell recruitment and migration along the blood vessels and nervous bundle, which conformed the dynamic compound structure and played important roles in acupuncture.

## 1. Introduction

Previous study has proved the evidence that nervous system [1, 2], blood vessels [3], and mast cells [4–6] have a relationship with acupuncture. However, there is still lack of evidence from the histology that these parts work as a whole. In this study, we find a new histological method with which we can simultaneously display the nervous tissue, the blood vessel, and mast cells in the same section of acupoint.

## 2. Method

### 2.1. Animal and Material

Twenty male Wistar rats were kept in an animal house maintained with a 12-hour light-dark cycle and were given free access to food and water. The weight of the rats was 120–150 g. The other instrument and materials are as follows: the vulcanization acetylcholine iodized salt (sigma-Aldrich, USA), Toluidine Blue O (Amresco, USA), constant cold box slicer (Thermo Fisher Scientific, UK).

### 2.2. Interventions and Histological Stain

The rats were separated into control group (Con) and electroacupuncture group (EA) randomly, in EA group, left Zusanli (ST36) and Weishu (BL21) were stimulated with electroacupuncture machine, and the parameters are 0.1 mA, 2/15 Hz, for 25 min every day, and the duration is 2 days. In Con group, there have been no interventions. The tissue samples including skin, subcutaneous tissue, and muscle tissue were collected from Zusanli and Weishu acupoint (size: 1 cm × 1 cm × 0.4 cm) in both EA and Con groups. Then the 15 *μ*m thickness section was prepared for double staining with Copper ferrocyanide method and acetylcholinesterase stain method. Finally, the section was dyed in 1% Toluidine blue solution for 15 second. The tissue section was observed with microscope.

## 3. Result

**Figure 1:**
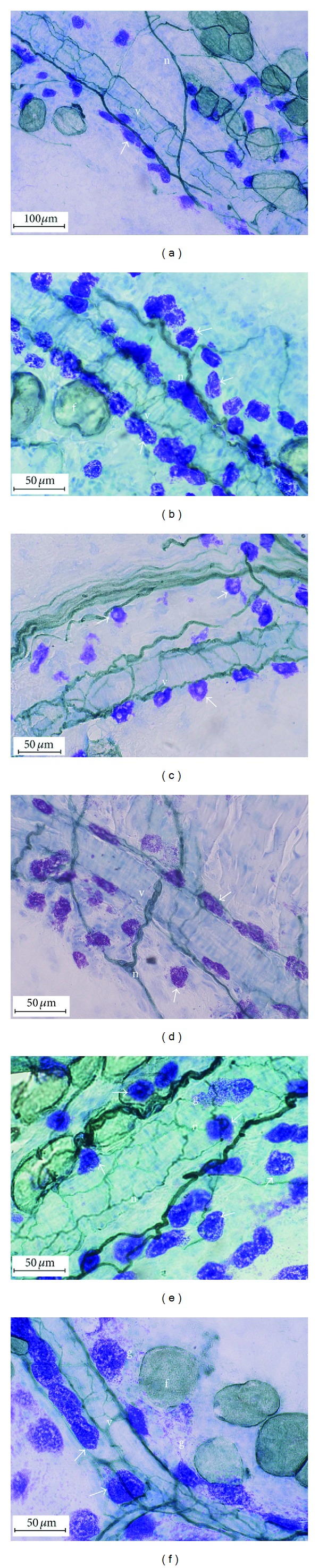
(a) Weishu acupoint in Con group. (c) Zusanli acupoint in Con group. (b) Weishu acupoint in EA group. (d) Zusanli acupoint in EA group. (e) Weishu acupoint in EA group. (f) Weishu acupoint in EA group. ↙: MC; v: blood vessels; f: fat tissue; n: Acher nerve fibers; g: particular matter released from the MC.

Under the microscope, the small and medium-sized artery in Zusanli acupoint area was longitudinal distribution along the limb long axis; the medium-sized artery in the Weishu acupoints was longitudinal distribution, too. However, the small artery and vein in the Weishu area were mutual anastomosis with each other and form the trunk long axis blood network. The blood vessel layers can be identified, with the smooth muscle distributed in the wall of the small arteries (diameter, 20–200 *μ*m) and the capillary tortuosity into the group. Cholinesterase-positive nerve fibers (Acher) with different thickness were clearly visible, surrounding the blood vessels in the arterial wall, which has the close relationship with the vascular smooth muscle (Figures 1(a) and 1(e)). There are thick cholinesterase-positive nerve fibers near the vascular form into the sparse network. In the dermis, subcutaneous and muscle tissues of the acupoint area, the different sizes and numbers of MC could be found. Most of these MCs array along the small blood vessels and neural network with a special sequence and come into a close contact with each other (Figures 1(a), 1(b), and 1(c)). Finally, a compound band structure constituted by MCs, blood vessels, and nervous fibers will be found in the acupoints (Figures 1(a)–1(f)). In the point area of Con group, the number of composite banded structure constituted by blood vessels, nerves, and MC is less sparse arrangement (Figure 1(c)). In the EA group, MC number and degranulation number of MC increased in the acupoint area (Figures 1(d) and 1(f)). In addition, EA stimulation not only resulted in MC's migration and recruitment around the blood vessels, but also caused the MC cell body increases and deformation movement, which provided the space and path for MC's array in a special order along the nervous blood vessels network. As a result, the space-time function structure constituted by the MC + blood vessels + nervous fibers obviously increased in the acupoint, and this special structure will extend to the adjacent acupoint area (Figure 1(b)). 

## 4. Discussion

Previous research suggested that acupoint is relatively dense structures such as nerves [7], blood vessels, lymphatic vessels [8], and tissue space area [9]; however, there is lack of morphological evidence of whether or how to contact with each other. This study indicated that in the acupoint, the MCs, vascular vessels, and nervous fibers formed the space-time composite strip structure, and the internal components of this trip structure have the close and reciprocity correlation. This strip structure is not limited to the acupoint area but extended to the adjacent area, which forms the web channel for signal transformation between somites [10]. This phenomenon can be explained by the traditional meridian theory.

Under the action of acupuncture, MC was migrated and recruited in the acupoint and meridians. As a result, the active components which come from vessels, nervous fibers, immunity system, and endocrine system interacted with each other to form a functional coupling structure. According to the properties of this composite structure and correlation with the acupuncture meridians effect, we defined this special structure as mobile cell communication web system.

Mobile cell communication web system mainly consists of the vascular network subsystem, neural network subsystem, the immune network subsystem, and the channel network subsystem. The vascular network subsystem is the source of human activity which collected the materials, energy, information, and also the orignal ditch of mobile cells. It is also the metabolism foundation of tissues and cells and stable physiological functions. 

Neural network subsystem is an important information control system, linked to various organs through different nerve endings. For example, different caliber blood vessels closely related to the different types of neural endings which had different neurotransmitters and peptides regulated the flow of the peripheral blood circulation precisely [11] then influenced the transport and distribution of body fluid and took part in the stress response and nerve-endocrine-immunity procedure.

Mobile cell subsystem is an active subsystem, which plays an important role in cell-cell or tissue-tissue communication through cell migration. In particular, the increased number of mobile cells or particle matters released from the mobile cell reflected the correlation and dynamic function of these subsystems.

Therefore, the mobile cell subsystem is an important order parameter in switching and regulating the acupuncture effect. The mobile cells take part in the cascade reaction of acupuncture through multi-channel and multi-target.

After acupuncture, the MC number increased in the fascial spaces or channel spaces around the blood vessels. The volume of MC was enlarged, and the large amount active compound was released; as a positive feedback reaction, the channel spaces gradually enlarged. The enlarged tissue space is a special functional pathway which will be beneficial for MCs migration in a special order, and finally, these special spaces formed the tissue channel as an important exchange pathway for material, information, and energy [12, 13].
